# Healthcare improvement as planned system change or complex responsive processes? a longitudinal case study in general practice

**DOI:** 10.1186/1471-2296-14-51

**Published:** 2013-04-23

**Authors:** Barbara J Booth, Nicholas Zwar, Mark F Harris

**Affiliations:** 1 Centre for Primary Health Care and Equity, School of Public Health and Community Medicine, University of New South Wales, Sydney, NSW, 2052, Australia; 2 School of Public Health and Community Medicine, University of New South Wales, Sydney, NSW, 2052, Australia

**Keywords:** General practice, Quality improvement, Complexity, Organisational change

## Abstract

**Background:**

Interest in how to implement evidence-based practices into routine health care has never been greater. Primary care faces challenges in managing the increasing burden of chronic disease in an ageing population. Reliable prescriptions for translating knowledge into practice, however, remain elusive, despite intense research and publication activity. This study seeks to explore this dilemma in general practice by challenging the current way of thinking about healthcare improvement and asking what can be learned by looking at change through a complexity lens.

**Methods:**

This paper reports the local level of an embedded case study of organisational change for better chronic illness care over more than a decade. We used interviews, document review and direct observation to explore how improved chronic illness care developed in one practice. This formed a critical case to compare, using pattern matching logic, to the common prescription for local implementation of best evidence and a rival explanation drawn from complexity sciences interpreted through modern sociology and psychology.

**Results:**

The practice changed continuously over more than a decade to deliver better chronic illness care in line with research findings and policy initiatives – re-designing care processes, developing community linkages, supporting patient self-management, using guidelines and clinical information systems, and integrating nurses into the practice team. None of these improvements was designed and implemented according to an explicit plan in response to a documented gap in chronic disease care. The process that led to high quality chronic illness care exhibited clear complexity elements of co-evolution, non-linearity, self-organisation, emergence and edge of chaos dynamics in a network of agents and relationships where a stable yet evolving way of organizing emerged from local level communicative interaction, power relating and values based choices.

**Conclusions:**

The current discourse of implementation science as planned system change did not match organisational reality in this critical case of improvement in general practice. Complexity concepts translated in human terms as complex responsive processes of relating fit the pattern of change more accurately. They do not provide just another fashionable blueprint for change but inform how researchers, policymakers and providers participate in improving healthcare.

## Background

Changing population demographics, evolving disease patterns and new research findings, exert unremitting pressure for change in health care, demanding reliable ways for continual improvement. General Practice, for example, which evolved in response to acute, episodic illness, must now respond to increasing prevalence of chronic illness and to research suggesting better outcomes when primary care is organized according to a chronic care model [1]. The quest for a quality improvement “magic bullet” [2] has been accelerating for 20 or more years and the source domain for effective change methods has broadened from education (undergraduate, vocational and continuing) to include social theory (peer influence and opinion leaders), cognitive approaches (guideline development) and organizational management theory (total quality management, system change) [3-5]. A number of policy-level “think-tanks” have been held and reported in major medical journals [6-8]. Even naming this area is contested: implementation science, adoption, quality improvement, dissemination, complex intervention and knowledge translation [9-11]. Yet the intense research and publication activity has not resulted in reliable prescriptions for success [12-14]. There has been little advance on the “modest to moderate improvements in care” observed in systematic review of different strategies for implementing change which led to the conclusion that there is an “imperfect evidence base” available to guide efforts for improving care [15].

This paper seeks to challenge the dominant discourse in this area of interest. We look at one common prescription for local implementation of best evidence and offer another perspective on how change might occur, then examine real-life experience to test both explanations. As we do this, we also seek to explore what can be learned by thinking within a complexity paradigm about change and improvement in the organisational context of general practice.

### *The dominant discourse in healthcare improvement: planned system change*

Implementation science promotes methods influenced by systems thinking [16], particularly cybernetic systems, developed in the 1950s: “self-regulating, goal-directed systems adapting to their environment” [17]. The first step is to focus on one area where there is evidence for better practice – the desired “state” for “the system” – and to identify any “evidence – practice gap” in structures, processes or outcomes [18]. The next steps identify existing and potential barriers and facilitators for change in the desired direction [19]; design and implement an intervention (simple or multi-factorial); and then monitor and evaluate achievement towards closing the “evidence – practice gap”. Plan-Do-Study-Act cycles [20] are one example of a small scale quality improvement technique based on systems approaches of organizational learning.

The thinking is linear, where multiple variables contribute differentially in measurable ways to determine a measurable outcome. It is also analytic, to explore these independent variables to build up a picture of what comprises “the system” and how these interactions work; and reductionist, by focusing on specific and measurable parts. Once identified and understood, component parts and their interactions are assumed to remain constant (controlled) while others are modified so that predictable results of interventions can be expected. This mental model leads to mechanistic ways of speaking. For example, Shojania and Grimshaw [21] talk about identifying factors driving provider and organisational change, while Leykum et al. [14] speak of designing interventions to leverage improvement. Overall, the goal is to incorporate research into routine practice in a timely and reliable fashion. The key elements of the method are design, control and predictability: understanding the design and changing it in controlled ways in order to achieve predictable outcomes. This is done by objective “managers” from outside “the system”.

### *How would a practice develop better chronic disease management using planned system change?*

Evidence for a better way of doing chronic illness care [1,22] would prompt an evaluation of the practice using an instrument such as the Assessment of Chronic Illness Care (ACIC) developed for this purpose [23]. Next, one or several clearly defined aspects of care would be chosen as the focus for change, based on explicit criteria such as seriousness of the gap, potential for health gains, political priority, or being more amenable to change. This bounded area of practice would be examined in detail to explore barriers and facilitators for change, looking at matters of staffing, information systems, culture, financial resources etc. Next would be planning one or a sequence of interventions, including specifying activities, personnel, timelines, accountabilities, measurable milestones and performance targets. Activities might include education (using a broad range of methods such as feedback and peer influence), financial incentives, new staffing and role changes, even sanctions. The final stage would involve implementation, with monitoring achievement of milestones and final evaluation of outcomes. The steps would be designed and managed from “outside” the system, whether by external change agents or researchers or members of the practice taking on an “objective planner” perspective.

#### *An alternative paradigm: complex responsive processes of relating*

Complexity concepts have become popular in offering a different view of organizational reality that is relevant to healthcare and how its pattern of organizing is changing within the broader sociopolitical environment [24-27], but are used in a variety of ways with more or less looseness in their application. Elsewhere, we have described in detail the fundamentals of complexity thinking in relation to general practice and primary healthcare organization, particularly core elements from the study of complexity in the natural sciences: non-linear dynamics; networks of agents and relationships; co-evolution; self-organisation; emergence; and edge of chaos dynamics [28-30] (see Additional file 1 for expanded explanations). At the same time we also critiqued loose translation of complexity concepts into human organizing, (such as self-organisation as empowerment, or edge of chaos as a pejorative description of poor management) and argue against the tendency to image organizations directly as complex *systems*. This merely offers new jargon for approaches based on the dominant discourse of organization as system, an *object* with properties represented by *variables*[31] Rather, we follow Stacey and others from the Centre for Complexity and Management at the University of Hertfordshire, who draw analogies from complexity sciences in the light of modern pragmatist sociology and psychology, in order to understand organisational reality as complex responsive processes of human relating [32-35].

The non-linear networks of agents and relationships that make up complex adaptive systems in natural sciences are, in human organisational reality, analogous to groupings of self-aware people (not *variables*), with their personal and social context, their current emotions and values, interdependent through continual involvement in communicative interactions with each other and broader society. Their relationships are paradoxically enabling and constraining at the same time, reflecting the power of social mores, hierarchies, politics and culture. They need not be harmonious. These relationships co-evolve, so that individuals and groups continually both influence, and are influenced by, each other. Self-organisation is not mediated by externally pre-set rules but is simply local interaction – processes of *communicative gestures and responses*, *power-relating* and *ideology-based intending, choosing and acting*. Order, what we commonly identify and name as “the organisation” (and, indeed, society as whole or part), emerges from these many, many local interactions. Just as the dynamic of richly connected complex systems tends to the *edge of chaos*, so complex responsive processes of human relating exhibit paradoxical qualities of stability and potential for radical transformation.

### *How would development of good chronic illness care in a general practice be understood as complex responsive processes?*

Importantly, there might be no clear blueprint with readily identifiable components or stages to match against the experience of change (though plans for change may well be part of conversation in the practice). It might be hard to identify discrete planning and implementing activity, let alone objective planners, who are located outside the network and who are observing, making changes and assessing impacts. Clear boundaries of the network of agents and relationships involved in practice-level change would be hard to define, since communicative interactions both within and outside the practice would influence the pattern of organizing. Both enabling and constraining relationships would be identifiable and seen to influence change. The whole would be difficult to sub-divide into clearly defined parts for study and manipulation in isolation from the rest. Instead, we could expect changes in one area to influence other areas, with potential for reinforcement or opposition providing examples of disproportion between cause and effect. There might be evidence of values-based choices that identifiably influence responses to communicative gestures, and so shape the pattern of working that emerges as the practice “organisation”. Change would be paradoxically predictable – it would occur in any dynamic network – but unpredictable – the trajectory and detail would be unknowable into the future, and only readily discernible with hindsight.

### *Purpose of this research*

In proposing complex responsive processes as a new explanation for how change might occur in general practice and primary health care, it is important to test how theory matches real life. We also need to explore whether this way of thinking and speaking offers useful insights in an environment of continual health care improvement and reform.

## Methods

### Research design

Exploring change from a complexity perspective has implication for research design, implicitly rejecting detached observation. The phenomenon of interest can best be investigated from within the action in ways that are inherently subjective and interpretive [36]. Further, a reductionist approach would restrict the scope of enquiry where the whole is greater than the sum of the parts and would eliminate co-evolution. Most research into healthcare improvement and organisational change makes claims for knowledge based on a post positivist stance [37], where causes are studied as determinants of effects and reduced to smaller subsets of ideas for testing. Objective measurement is crucial and the purpose is to develop, test and refine theory. Exploring how complexity thinking might inform our understanding of organisational change demands different claims to knowledge. This study takes a pragmatic and constructivist approach which seeks understanding of everyday reality from multiple participant meanings.

We used the qualitative approach of case study, “an investigation of a contemporary phenomenon within its real-time context, when the boundaries between the phenomenon and context are not clearly evident and multiple sources of evidence are used” [38]. The case was instrumental in providing insights into an issue, [39] in this instance organisational change for better chronic disease management in general practice, and the purpose was to explore the single case as a critical case to challenge the dominant discourse. This purpose was served by using pattern-matching logic [38] to compare an empirically based pattern with two alternative predictions (planned system change or complex responsive processes) defined prior to data collection and articulated in the Background section of the paper. We determined that the phenomenon of interest – organisational change – required examination at both local and national levels of analysis, so used an embedded, single case study design. This paper presents the results of the local level of the case. Given this rationale, selection was determined by the potential for learning rather than representativeness [39] according to two simple criteria: that the practice deliver good chronic illness care, without being markedly atypical in size, location, history or funding. Sampling was therefore purposive and convenience based through word of mouth. A mid-sized practice with a reputation for well-established chronic illness care was recruited and visited over a six month period in 2007.

### Data collection and methods

**Figure 1:**
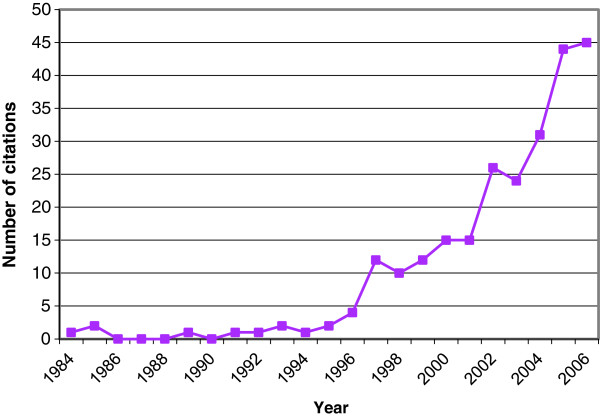
Medline citations using “chronic disease management” as keyword 1984 – 2006, search conducted 16 February 2007.

Multiple sources of data were used to examine the quality of chronic illness care and to explore the understanding of participants in how this evolved. Purposive sampling was also used to determine the period of interest for the case, when awareness of an evidence-practice gap in chronic illness care might arise. Medline was searched for articles using the term “chronic disease management” and annual counts made to determine the period over which it became prominent (Figure 1). Government initiatives during this time were tabulated from Australian Government Department of Health archives to provide a clear policy context [40,41] (see Additional file 2). In-depth, semi-structured interviews were conducted with all practice staff (Table 1), guided by an outline covering a description of the practice, how it was organized, and how it had changed in the period of interest, particularly with respect to organisation of chronic illness care. As well there was direct observation over multiple visits of work processes, facilities and interactions, and two practice meetings were observed and recorded. All recordings were transcribed in full. Practice documents (accreditation reports, policy and procedures manual, recall register, appointments schedules) were also examined.

**Table 1:** Staff of the practice at the time of the case study: their roles and characteristics

	**Role**	**Age**	**Gender**	**Length of service**
P1	Partner	>=40	male	>10 yrs
P2	Partner	>=40	male	>10 yrs
P3	Partner	>=40	female	>10 yrs
A1	Associate	>=40	female	>10 yrs
A2	Associate	>=40	female	>10 yrs
A3	Associate	>=40	female	5-10 yrs
R	Registrar	<40	female	< 5 yrs
BM	Business Manager	>=40	male	< 5 yrs
PN1	Practice Nurse	>=40	female	5-10 yrs
PN2	Practice Nurse	>=40	female	< 5 yrs
OM	Office Manager	>=40	female	< 5 yrs
R1	Receptionist	<40	female	< 5 yrs
R2	Receptionist	<40	female	< 5 yrs
R3	Receptionist	<40	male	< 5 yrs
R4	Receptionist	<40	female	< 5 yrs

### *Data analysis*

The ACIC provided a descriptive framework to validate selection of the practice and to identify aspects of good chronic illness care whose evolution warranted exploration. Pattern matching logic was used to compare the reality of how change occurred to the two rival theoretical propositions outlined earlier in operational terms: planned system change or complex responsive processes. Transcriptions, field notes, documents and photographs were entered into N’Vivo qualitative research software (QSR International) for exploration in depth. Basic tree codes were developed for the rival explanatory frameworks (planned system change and complex responsive processes). Transcripts were coded using these tree codes plus extensive free coding of any concepts related to change and chronic disease care at the practice. These were consolidated and merged into the existing and two new tree codes – pattern of change and chronic disease management. Initial coding was done by one author (BJB) and samples were reviewed by and discussed with MFH and NZ.

### *Ethics*

The study was approved by the University of New South Wales Human Research Ethics Committee. All participants gave their informed consent to participate.

## Results

In order to protect confidentiality, some practice and personal details have been changed where they are not crucial to the exploration of the case study. Initially, we describe the practice and how chronic illness was managed at the time of the study – “the present”. We then examine the “past” 23 year story of the practice, focusing on the development of the elements of good quality chronic illness care. Finally, we return to the “present” of 2007 and explore the participants’ understanding of how change occurred/occurs and describe current patterns of interaction at the practice.

### The practice and chronic illness care: the present

The practice is located in an inner city suburb with a demographic of ageing working class immigrants and recent influx of younger middle class professional families. It has five full-time equivalent GPs (three of whom own the practice in a legal partnership), one full-time equivalent practice nurse, a part-time business manager, full-time office manager, and two full-time equivalent reception staff. All staff consistently identified core values of the practice – good quality care, ethical professional practice, and patients come first – while also judging it to be a democratic and friendly place to work. They saw clearly that these values outweighed financial interests, but acknowledged that this had been a source of tension at times.

Chronic illness care according to key elements of ACIC is an *explicit priority focus* at the practice. The doctors are aware of the burden of illness in their ageing patient population and describe how these patients need new ways of working, such as risk management, planned care and follow-up, and patient self-management. Practice nurses play a key role as care coordinators. They have their own appointments, conduct health assessments, help to prepare care plans, maintain registers, arrange reminders and conduct reviews. They also undertake preventive, clinical and organisational tasks.

Financial and *administrative arrangements support chronic illness care*. The business manager (BM) promotes use of Medicare items for chronic illness care and works with the office manager and nurses on appropriate care processes. The different members of the practice team have a reasonably clear and shared understanding of their own and each others’ roles, which generally corresponds to the organisational chart in the practice manual, with the three partners clearly in the senior management role. Arrangements for delivery of care are reviewed as needed at the monthly practice meeting.

The practice doctors *utilize community resources*, public and private, to provide multi-disciplinary care. There is an intentional process to maintain awareness of the role and quality of such community-based services, including visiting new services and discussion at the practice meeting. Practice members participate in their local Division of General Practice (indeed, PN1 is used by the Division as a resource for education about chronic illness care) and numerous professional networks.

Doctors and nurses work together to provide education *to help patients understand and participate in their own management*. Practice staff describe a strong culture of patient-centeredness and clinical staff emphasize the need to engage patients with chronic illnesses in learning how to participate in their own care (although with variable success). The doctors are aware of a broad range of up-to-date *guidelines for management of chronic illness*, although have some reservations about the plethora of materials and the robustness of the sources. They all participate in continuing professional development. The practice meeting is explicitly used to share individual learning and the meetings observed revealed both wide knowledge and critical appraisal in the area of chronic illness care.

The practice uses a blended paper and electronic medical record system, with a *register of patients with chronic illness* used to provide patient reminders. At the time of data collection, they did not routinely evaluate their chronic illness care through regular record audit. Two principals (P1 and P3) were aware that this was desirable and occurred in other practices.

Within the Australian context [42], this level of development in all aspects of the Chronic Care Model validates the practice’s reputation for good quality chronic illness care.

### Chronic illness care: how it developed

In this section we present both a chronological history of change within the practice and exploration of how and why it happened this way from the memories and interpretation of the participants involved in the action. Table 2 shows a detailed timeline of key developments at the practice correlated with key developments in the policy environment of general practice in Australia.

**Table 2:** The story of the practice

**Practice history**	**Year**	**Public context**
P1 buys into practice	1985	
	1986	
P2 buys out remaining partner	1987	
	1988	
	1989	
P3 joins as associate, then partner	1990	
	1991	
	1992	GP Strategy
	1993	Better Practice Program, Divisions of GP, GP Research Program
Move to new premises	1994	
Record audit	1995	
Ceased bulk billing	1996	
	1997	Immunization strategy
PN1 starts	1998	
	1999	Enhanced Primary Care (EPC)
Accreditation	2000	
	2001	Asthma 3+ Plan
	2002	
Re-accreditation	2003	“Red Tape” report, Medicare Plus, nurse rebates, simplified EPC
BM starts	2004	
OM starts	2005	
	2006	
Case study	2007	

#### *Foundation of the practice: the first ten years*

In 1985, P1 bought the practice, selecting this one among many because of its ethos that earning capacity was not the primary consideration. P2 and P3, sharing the same values, joined over the following five years and the partners acquired, renovated and moved into purpose designed premises in 1994. P3 came from an overseas medical school with a progressive primary care program, and was seen as an agent for change. The initial joining of the three principals, partly by chance but with shared values, led to a continuing intention towards delivering the best quality of care in the practice, which made them open, even eager, to change:

P3: the theme of change and improvement … has always been there… I mean P1 and P2 … if they knew there was a better way, they wouldn’t actually choose for conservative, to stay the way they are. If there was a better way, they would go the better way.

PN1: they like to be seen to be a bit more cutting edge … They like to be up front and like to be seen to be progressive…

BM: …a very strong values system … that was non-negotiable … always at the cutting edge of doing things differently … this place was always leading the charge.”

The fact that P3 had trained overseas meant that she had no existing referral networks among the local specialists, so she visited them to help establish her in a new place. As a consequence, however, she thought that this brought her (and the practice) to notice and meant that they gained a reputation as interested and progressive, leading to her being approached to participate in a quality improvement research project that was an early initiative in Australian general practice reforms.

#### *“Lots of things changing”: the next five years*

This research project was seen by all three partners as highly significant – each referred to it when asked how the practice had developed into a leader in chronic illness care. It came not long after the move in 1994, among many other memorable changes, some related to the research project – directly or tenuously – and others apparently unrelated. The research involved a record audit of preventive care, including Pap smear, immunization and HbA1c. The results revealed rates lower than their anticipated excellence and this disturbed the partners. Having identified suboptimal care, the project explored possible remedial actions, particularly setting up recall and reminder systems. This played out differently in each of the three preventive care areas.

Setting up a Pap smear register and reminder system proceeded reasonably smoothly. Cervical screening was topical and an area of interest for P3 and the research team, and a way forward seemed clear:

P3: so ‘94 we didn’t have computers. Reflecting on what we had to do we had to have computers

So, in 1996, the practice introduced computers for clinical work, well before the 1999 Australian Government incentives for electronic management of clinical information in general practice.

The response to diabetes management through HbA1c testing was a different story. Although P1 reported a personal revelation about the different requirements for managing patients with chronic illness, there was little change in the pattern of how the practice delivered their care.

P1: I can give you the major change … the agenda of the consultation [in chronic illness patients]

Q: What happened? One day did you just think “Hmm, there’s a lot more chronic illness, I’ve got to have my own agenda when patients come in”?

P1: Yes! There was actually a revelation … it’s that sort of dis-ease, the discomfort that you live with when you think that you’re not doing things well

Q: Mmm, so how does something like [planned care] fit into that …was all that changed? – you just saw things in a different way?

P1: Yes!

Q: Did it just work immediately?

P1: No! (laughter).

The practice did, however, take action towards the end of this period on the third focus area in the research project – immunization – but this was in response to other influences in addition to the audit. At the same time as there was growing tension in the practice about financial matters (*P2: …we got fed up with, um, constantly feeling like we were battling to make any kind of living …practicing the sort of medicine we did …*), the Australian Government introduced a new immunization strategy that included incentive payments for general practice as well as social marketing to encourage immunization. At the practice, improving immunization became important both to re-affirm their value of leadership in quality of care and, at the same time, to provide some relief from their financial stress. They also felt that the task might not be too difficult, since the issue might be in the recording, rather than actual immunizations delivered.

#### *The first practice nurse*

As a consequence, in 1998, PN1 joined the practice, initially to update the immunization records. The employment of PN1, a senior nurse with some hospital management experience, was seen by P1, P2 and P3 as pivotal to many of the changes that subsequently led to better chronic illness management. However, there were slightly different interpretations from each of the principals and from PN1 herself as to how this came about. She was, in fact, the wife of P2, and according to him, flexible, part-time work at the practice was an ideal opportunity for her to “*get out of the house*” [P2]. It was a fortunate coincidence that someone who understood medical terminology and could find the way around a medical record was available to help update the immunization data. And then one thing led to another:

PN1: …it was then, oh no, no, we don’t want a nurse. And then … do you know how to work the ECG machine? oh yeah, I can do that. … oh do you want to work another day? oh, well, you know, all right, for a few hours… then what’s this Medicare change? I’ll read it and I’ll let you know. So my role sort of went from just doing the immunisations until … making sure things were done for PIP and then accreditation came and it was like, do you know what this means. And I said I’ll give it a go

However, PN1’s gradual increase in responsibilities was not easy for some of the doctors.

P1: I remember distinctly, when PN1 started, and, she started to do more I was very resentful. It was a huge issue for me and I’m sure for a couple of the other doctors of letting go. You know?

#### Enhanced primary care and accreditation

As practice accreditation was gaining momentum, the Australian Government launched the Enhanced Primary Care (EPC) Program that provided insurance rebates for planned chronic illness care – health assessments, case conferencing and written care plans – outside the traditional fee-for-service structure of episodic, reactive, time-based consultations. PN1 was invaluable as the practice decided to undertake its first accreditation and began to work out how to use the new item numbers. Being one of the first practices to be accredited was consistent with the practice’s “ahead of the pack” culture:

Q: How did you decide to get accredited? One of the first practices…

P2: I think we just felt like it was our duty to do it yeah, I don’t know. Well it was tied up with PIP payments and all that kind of thing as well so we thought, good practices do it, we should you know, maintain some sort of objective standard I suppose.

Accreditation was a key turning point for both PN1 and many of the processes for chronic illness care. It gave PN1 a pivotal and important role, and it involved considerable “tidying up” of existing processes. It was disruptive and met with some resistance, but the end result was a sense that the practice had gained quite a lot and PN1 was secure in a valued role for helping make changes happen smoothly in the practice.

Q: who … brings things in from the outside or comes up with new ideas?

A2: PN1, … she gets a lot of information sent to her on new things …through the [local Division of General Practice] …

The practice was now well placed to respond to further government refinements of the EPC program, such as incentive payments for achieving more steps in the cycle of care for chronic illness and including mental health within the program. The next four years were ones of incremental changes, refining the use of EPC and easily navigating the second accreditation cycle.

#### Attending to business

Despite this, the practice remained under financial pressure during 2000 – 2004. In particular, P3, as a female GP with an interest in women’s health, tended to have longer and more complicated consultations, but these did not receive proportionally higher Medicare rebates, limiting the fees that could be charged.

P1: P3 more than all of us, … was making the least amount of money for the effort she was putting in, … but all of us had noticed that our incomes had not done very well in the previous 3 or 4 or 5 years and P3 was the one who was feeling it most…

P3: you know it wasn’t sort of working for us financially, and that was a bit uncomfortable, …We were actually not making money, … you know, here you are working day in, day out …

As a consequence, the practice brought in a small business consultant, the cousin of P3’s husband, to review the financial situation. His analysis identified some ways to improve cash flow, but he also suggested more extensive changes to work processes and staffing to improve business viability and efficiency. He was subsequently engaged as an external, part-time business manager. He negotiated new remuneration arrangements with the GP associates and reviewed how the front office worked, encouraging more responsibility among the receptionists. His stated aim for both was to change the management style to improve teamwork:

BM: the non-clinical staff – there was all care, but no empowerment … and the big change I’ve had is … to empower the staff to become more involved and … seeing themselves as … a critical part of the whole team from start to finish.

It was a time of rapid change and discomfort, both for staff and the partners.

BM: when I first came in, to introduce those changes, they happened in a very short period of time and there was a lot of pain about that and there was a lot of reluctance [by partners] … to let go of decision-making…

Subsequent steps were to appoint an office manager and second practice nurse, and to more clearly delineate their roles to free the practice nurses from administrative tasks. This allowed greater priority to be given to chronic illness care, with more intentional and systematic use of the EPC items, which carried significantly higher rebates. The office manager and practice nurse then worked together to refine processes to make this new staffing structure work.

OM: the staff out the front … they didn’t know what all these things were and that, … care plans sometimes took 45 minutes or an hour …It was chaos, it was chaos, … so yeah it was like “no hang on a minute, we’re just getting into a mess here”.…it wasn’t like a formal meeting, it was more just “PN1 have you got 10 minutes?”, you know, “this isn’t working”.

And the process seemed to be successful.

BM: So that’s a significant change … you can actually …empower and give them the tools to make their decisions, … then you start to see the improvements. You …start to see productivity increase and you start to see happier people and we’ve got a very happy workforce here.

A3: … at the time we were all really cranky. I was really cranky, I was you know about to leave really cranky you know that sort of thing. And now I’m really happy, …

R3: Everyone here sort of tries generally speaking to do their best to … cooperate in getting what needs to be done, done.…I did work for another medical practice before this one … here … we work with the medical staff rather than for them.

At the end of this period, the practice was effectively in the form it was during the case study.

### Participants understanding of change

The participants struggled to explain how their good chronic illness care came about. They all described plenty of change, but no-one could readily point to a planned, targeted strategy for chronic illness care (although there were several examples of planning, trialling and implementing improvements for more discrete problems, such as handling pathology results). Some (A2, R1 and R2) seemed content to focus on their personal story and how things worked in the present. Most skipped about within the history of the practice and across clinical areas, making connections and identifying key events or turning points in the way chronic illness care developed. BM related a process of planning and change to bring financing and staffing more in line with modern business practice, and this had a significant impact on chronic disease management through increasing use of the EPC items. Financial incentives were important but not as a simple lever: there were several nuanced understandings of how they influenced change.

P1: I think that by far the biggest force for change has been money, you know … incentives, … So if somebody says you’re going to get and extra $300 to achieve 98% immunisation rate rather than 95%, I think the $300 is not all that important but it becomes an interesting exercise to see if you can achieve it. Because you know that’s an area that you should be going, because it’s an important thing to do, so if somebody else recognizes that it’s an important thing to do I think…umm …it can drive it in chronic disease management.

Q: So why did you decide to get involved in EPC in the first place then?

P2: Um I don’t know, well I can personally see the advantages, … it um sits well with me ideologically that the government is trying to do chronic care properly and, and de-emphasise the acute reactive kind of medicine. So for a start I thought the principles were fine.”

A3: …actually getting the nurses involved in that process, … and able to … write a sensible care plan ….

Q: So who made that happen?

A3: BM essentially …because it was a money making exercise … I mean it’s useful for us because care plans are a useful thing. But I think it was a financially driven decision in many ways.

The financial incentives allowed for new way of organising.

Q: Did the money make a difference?

P1: Yes! … not so much for the income but for the fact that you feel you can support the appointment of a nurse. That’s become important. … I mean, virtually all our money goes into the employment of a nurse, that we get from those extra items, …umm, but that’s useful, yes.

### *Change in action: communication and planning*

The dynamic of the practice meeting revealed the pattern of organizing that fostered change and improvements over the years. Matters were discussed according to an agenda that began to be defined when the practice meeting was scheduled. A dedicated space on a whiteboard in the staff room was left for the meeting agenda, open to any staff member to add an item they felt at the time was important to discuss. At the meeting, the person who had added the item opened the discussion, and others joined in as they wanted. There were no formal minutes, but if an item related to a problem, and it remained unresolved at a subsequent meeting, it was raised again for further discussion. Everyone at the meetings spoke freely and were listened to with interest and respect, and clearly accepted that some matters would be decided at the subsequent partners’ meeting.

During the 58 minutes of one practice meeting the following matters were dealt with: lunch; personal and collegial networking; administrative matters; clinical organising around a new government screening program; review of new community linkages; discussion of clinical information from continuing professional development; finance procedures; and prescribing audit results. Although improving chronic illness care was nowhere explicit, the discussions attended to community resources, management guidelines, reminder processes and care outcomes across a range of chronic conditions.

## Discussion

### *The pattern and process of change*

The overall pattern of change at the practice does not reflect planned system change, either through incremental continuous quality improvement nor the episodic transformation that might be anticipated from wholesale redesign. Rather, it resembles a punctuated equilibrium, more consistent with the power law of edge of chaos dynamics where small changes occur frequently, larger changes more rarely. In retrospect, it was possible to identify key times of change and the influences at work at the time. Apart from the Pap smear register and staffing review, there was no obvious architect nor blueprint for much of the change that occurred. But it was not simply random. It emerged in the interplay of intentions, communicative gestures and responses, power relating and values-based choices and actions of the partners, practice staff and policy makers in a range of areas, including chronic illness care.

### *How does the dominant discourse of planned system change match the story of the practice as it improved its chronic illness care?*

The research audit clearly identified evidence–practice gaps that stimulated improvement activity, with prioritisation of Pap smears according to interest, political priority and amenability to improvement. There was analysis of underlying causes and a planned way forward – computerisation and establishment of registers. There was considerable influence from the external research team, which was able to provide both objectivity and resources. Similarly, financial viability was identified as a need, particularly for P3, and that stimulated clear, planned improvement activity. BM, external to the practice, facilitated change by identifying contributing factors, then formulating and executing planned changes in staffing, remuneration and office procedures. BM continues to sit largely “outside” the practice in a part-time, off site role, providing review of financial data and processes, with the ability to intervene through regular meetings and discussions. Both these change processes occurred in areas that were quite easily “bounded” – they could be analysed and altered without too much interference from other aspects of the practice (even though both had considerable flow-on effects to chronic illness care). Other parts of the story fit the dominant discourse model less well. The response to the audit results for immunization rates, for example, involved rectifying a recording problem rather than strategies to improve immunization rates, although this flowed on to have profound effects for chronic illness care. In contrast, the specific results that showed poor chronic illness care, sub-optimal HbA1c recording, made the partners want to respond and improve, without any specific planning or improvement activity resulting.

### *How is the story of the practice understood as complex responsive processes of human relating?*

*Co-evolution* is an important complexity element in the story. Key aspects of good chronic illness care developed as a consequence of efforts in unrelated areas – both clinical and business. Robust linkages with community services developed in large part because a new doctor in the practice felt the need to establish a referral network to manage patients’ episodic care. An unrelated consequence was involvement in the research audit, which did not stem from a specific plan for improvement. Computerisation and capacity for patient registers arose initially from efforts to improve Pap smear rates. Electronic records and registers were refined in seeking better data on immunization to qualify for incentives, which also led to employment of a practice nurse, whose role and status grew through her management of accreditation. The need to improve financial viability through modernising business practices led to re-structuring administrative and financial processes to give priority to chronic illness care.

*Non-linearity* is also apparent. Participants themselves could not identify a clear pathway leading to improved processes for chronic illness care. Yet they clearly remembered a will and intent for change in this and could, in retrospect, identify the contributing influences to their current state. Sensitive dependence on initial conditions is evident from the pervasive influence of the foundational values of the practice partners, which have remained clearly articulated and understood throughout their story. As Crabtree et al. also concluded from their 15 year program of research [27] another practice in similar demographic setting with similar size and makeup could clearly take a very different trajectory over 25 years’ of evolution. Disproportion between cause and effect is demonstrated in the profound impact on chronic illness care from hiring a practice nurse to clean some data and provide convenient employment for a partner’s wife at a particular stage of family life.

*The network of agents and relationships* of which the practice is part is somewhat difficult to define, with communicative interaction between staff at the practice but also with local, regional and national general practice institutions. Policy initiatives also form part of the communicative interactions to which members of the practice are responding. In complex adaptive systems, the richness of relationships is an important factor in movement to *the edge of chaos* where transformational change and *emergence* of new order become more likely. *Power-relating*, both constraining and enabling at the same time, was evident in the paradoxical tensions felt within the practice by the employment and expanding role of the practice nurse. P1’s response to a perceived threat may well have been constrained by PN1’s relationship to P2, opening up potential for PN1 to take on new roles and responsibility. Similarly, there was tension in the circumstances of employing BM and the changes this brought, but the constraining needs of P3 and her relationship with BM may have facilitated this rather intrusive and difficult period of change. As well, the ethos of democracy communicated by the partners intentionally facilitates open communicative interaction among all staff.

*Ideology-based choice* was evident as an important influence in *self-organisation* in two main areas. Firstly, the response to the research audit results reflected the value of seeking to be at the forefront of quality. The response of doctors in other practices without this ethos might well have been denial or indifference. Secondly, the attitude to financial incentives is informative. Both P1 and P2 interpret the financial incentives in government policy initiatives as “communicative gestures”. They are powerful gestures, carrying significant advantages for the practice, as acknowledged, somewhat equivocally, by the partners. But they also communicate commitment and government values, which appeals at another level. The response of the practice partners is to respond to the values-based communication, while appreciating the benefits largely realised by the business manager’s somewhat different response. Other doctors, in other practices, with different values, might respond quite differently.

## Conclusions

This empirical comparison of the everyday reality of long-term change, in which one general practice developed good quality chronic illness care, confirms the conclusion of Suchman [43] that the dominant discourse of planned, stepwise change in strategically targeted areas of practice activity provides an inaccurate explanation of healthcare improvement. Complex responsive processes of relating, where communicative interaction, power-relating and ideology-based intending, choosing and acting produce patterns of organizing that are paradoxically stable and changing, helps to make sense of the evolution of the practice in ways that were not random, but also not according to a conventional linear blueprint for improvement. However, these different understandings of change are not an either/or dichotomy, as even the analytic method of pattern matching logic would suggest. Both are visible and not mutually exclusive in the change and improvement in this practice.

This study looks at organisational change for healthcare improvement at the practice level over a longer time frame than most empirical studies. This did not appear to challenge the recall of participants and was sufficient to discern the pattern of change to test the common prescription for how improvement should occur. The depth of exploration in all its particularity also reveals commonalities to help in broader understanding and learning.

### What are the implications of understanding organisational change as complex responsive processes of human relating?

At the local level, those in general practice who strive to respond to constant calls for improving care may find both reassurance and encouragement from the complexity-based conclusion of Westley, Zimmerman and Patton [44] that innovation “demands simultaneously that we set a course, move to action and relinquish the idea that we can control the outcome” (p223). Reassurance, since the complexity lens validates real efforts that did not seem to reach the desired goal; encouragement, because non-linear dynamics always hold the potential for transformation. Understanding co-evolution acknowledges the need for flexibility of improvement plans in response to everyday reality, at the same time promoting awareness of both collateral benefits and unintended consequences. The inherent unpredictability of the trajectory of future change focuses attention on ethical dealing in the everyday present, which should not be subordinated to uncertain future goals.

Leaders and researchers need also to reflect on such implications. As they plan and act to foster healthcare improvement, they are simultaneously “in control” and “not in control” of general practice [45]. There is no suggestion to abandon planning, policy-making or researching, but there could be benefits in a shift in emphasis to allow more tolerance for local adaptation. Evaluation should take account of collateral benefits, unintended consequences and what has been learned along the way, not only enumeration of discrete achievements along a pre-specified trajectory. Time lines may need to be longer to allow for co-evolution and the multiple attempts and circuitous routes that non-linear dynamics suggest. Drawing analogies from complexity sciences and interpreting them through modern sociology and psychology as complex responsive processes of relating can enrich understanding of policy and funding initiatives. They are communicative gestures that will evoke a wide variety of responses, based on power relationships and values-based choice, in general practices across the country. Ensuring and making clear the alignment between principles and purpose of the gesture, and its inherent power (such as financial incentives, regulations or sanctions), may influence the responses from which will emerge changing patterns of organizing.

A complexity perspective inherently precludes outlining any alternative prescription for implementing research findings in general practice to replace the dominant discourse. It does, however, suggest shifts in thinking and speaking about how health care in general practice might improve to meet changing needs and research findings. The current discourse can lead to frustration with the lack of anticipated progress and escalating intensity to seek ways to ensure certainty of outcomes. Understanding and learning from a complexity perspective helps to make experience of healthcare improvement more intelligible and less anxiety-provoking for leaders, researchers and participants.

## Abbreviations

A (1 2 3): Associate doctor at the practice; ACIC: Assessment of Chronic Illness Care; BM: Business Manager at the practice; EPC: Enhanced Primary Care; OM: Office manager at the practice; P (1 2 3): Partner doctor at the practice; PIP: Practice Incentives Program; PN (1 2): Practice nurse at the practice; Q: Questions by interviewer; R (1 2 3): Receptionist at the practice.

## Competing interests

The authors declare that they have no competing interest.

## Authors’ contributions

BJB designed the study, carried out the practice visits and interviews, analyzed the data and drafted the manuscripts. NZ and MFH participated in the design of the study, reviewed coding and data analysis and critically reviewed sequential drafts of the manuscript. All authors read and approved the final manuscript.

## Pre-publication history

The pre-publication history for this paper can be accessed here:

http://www.biomedcentral.com/1471-2296/14/51/prepub

## Supplementary Material

Additional file 1 **Expanded explanations of key complexity elements from natural sciences**[31-33]. Click here for file

Additional file 2 **Key Commonwealth (National) Health Policies relevant to chronic illness care in Australian General Practice**[40,41]. Click here for file
