# Supplementary material for: Healthcare improvement as planned system change or complex responsive processes? a longitudinal case study in general practice
Source: BMC Fam Pract. 2013 Apr 23;14:51. doi: 10.1186/1471-2296-14-51 (PMC3644498; doi:10.1186/1471-2296-14-51)
Supplement: Additional file 1 — Expanded explanations of key complexity elements from natural sciences[31-33]. [file 1471-2296-14-51-S1.docx]

**Additional file 1: Expanded explanations of key complexity elements from natural sciences [47, 48, 49]**

##### Non-linear dynamics

Dynamic systems exhibiting linear relationships, where the whole is equal to the sum of its parts, which act independently of each other, are easy to model mathematically. One or more parts can be held constant to examine other parts in isolation, and small perturbations tend to be damped down throughout the system, so calculations do not need to account for very small variability. In systems that exhibit non-linear dynamics, the whole is greater than the sum of the parts and mathematical models require computer simulation. Such systems exhibit *sensitive dependence on initial conditions* and small perturbations can be amplified throughout the system. Examples from natural sciences are found in weather patterns, molecular biology and ecology.

##### Network of agents and relationships

The focus across complexity thinking is on agents and their network of relationships – constraining and facilitating, single and multiple. One example is a Boolean network, which can be studied mathematically but can also be visualised easily as light bulbs in various arrays with interconnections that determine whether bulbs are on or off. There are various numbers of agents (light bulbs) and relationships (electrical connections) and natures of relationships (whether the connection turns a bulb on or off). Typical networks in natural sciences can include genes, ecosystems, neurones or economies.

##### Co-evolution

In such a network, each agent is continually acting and reacting to what other agents are doing. They do not exist or change in isolation, but co-evolve. The state of each light bulb switches, and is switched, on or off by the state of those around, producing different patterns of lights. Another example is an ecosystem. An animal’s ability to survive and thrive depends on its niche, determined in part by other animals in the environment, and their impact on both the original animal and the environment. None of these is static. As one animal or population changes, so others react, changing the environment and causing pressure for further change.

##### Edge of chaos

Experiments with Boolean networks revealed that sparse numbers of connections between agents create a system where co-evolution rapidly reaches stability, but in very densely connected networks, co-evolution leads to chaos (in the light bulb image, apparent random blinking of bulbs that never settles down into any observable pattern). In between, co-evolution produces coherent patterns that propagate, grow, split apart and recombine in an orderly way. This leads to the concept of the *edge of chaos*, poised between rigid structure and chaotic disorder, where there is also complexity: paradoxical stability and continuous change at the same time.

##### Emergence

The appearance of complex but coherent, patterned behaviours from simple, local rules is termed *emergence*. Another example is the flocking behaviour of birds, which has been simulated in computer modelling by giving artificial “boids” just 3 rules: maintain minimum distance from other objects in the environment, including other boids; try to match velocities with other boids in its neighbourhood; and try to move towards the perceived centre of the mass of boids in its neighbourhood.

##### Self-organization

*Self-organization* is the fundamental process of local interaction underpinning *emergence*. A further example provides important insights. Dribbling sand onto a table forms a pile whose shape remains constant over time, even as sand continues to dribble onto the pile. Each grain of sand is held in place by gravity and friction, poised such that another grain of sand dropping onto it may have no effect, may displace just the one grain, or several, or a cascade. The overall shape of the pile is maintained by local interaction, and all these responses occur at different times, following a power law where small displacements occur frequently, large avalanches much less often.
