# Supplementary material for: Healthcare improvement as planned system change or complex responsive processes? a longitudinal case study in general practice
Source: BMC Fam Pract. 2013 Apr 23;14:51. doi: 10.1186/1471-2296-14-51 (PMC3644498; doi:10.1186/1471-2296-14-51)
Supplement: Additional file 2 — Key Commonwealth (National) Health Policies relevant to chronic illness care in Australian General Practice[40,41]. [file 1471-2296-14-51-S2.docx]

**Additional file 2: Key Commonwealth (National) Health Policies relevant to chronic illness care in Australian General Practice**

***Medicare***

Medicare is Australia’s publicly funded, universal health insurance scheme that provides rebates to patients for services billed by private general practitioners according to a government schedule of service items – the Medicare Benefits Schedule.

***General Practice Strategy 1992 – 1996***

Successive budgets provided funding for programs in areas of Quality, Workforce, Integration and Financing, which included:

- Vocational Registration (VR) of general practitioners (introduced 1989)
- Accreditation of general practices
- General Practice Evaluation Program (GPEP): grants for research and evaluation in general practice to improve quality of care
- Divisions of General Practice (DoGP): locality-based organizations to foster integration of health services
- Grants for trials of co-ordination of care
- Better Practice Program (BPP): financial payments to supplement fee-for-service for defined services

***National policy initiatives 1997 – 2007*** [44]

1997 Immunization strategy

1998 Review of General Practice Strategy

1999 Replacement of Better Practice Program with Practice Incentives Program (PIP), with incentives for information management, after-hours care, rural and remote practice, clinical performance targets

1999 Enhanced Primary Care (EPC) item numbers added to the MBS targeted at better care for chronic illness

2000 self-management initiatives, both as part of EPC and demonstration grants

2001 funding for DoGP to support care for chronic and complex conditions; new Medicare items for chronic and complex conditions, Asthma 3+ visit plan

2003 Productivity Commission Report to simplify PIP and EPC, Medicare Plus restructure including rebates for services by nurses in general practices

2004 access to Medicare rebates for allied health under EPC

2005 diabetes & asthma service incentives and Better Outcomes in Mental Health program

2005 EPC simplified in response to “red tape” report with new items for chronic illness care

2006 EPC extended with further items and conditions

2007 Medicare items for geriatric assessment
